# Multidimensional Benefits of Weight Management in Old Age: The Mobility and Vitality Lifestyle Program

**DOI:** 10.1093/geroni/igaa057.3078

**Published:** 2020-12-16

**Authors:** Steven Albert, Elizabeth Venditti, Barbara Nicklas

## Abstract

The high prevalence of overweight or obesity in older adults is a public health concern because obesity is associated with risk of mobility disability. The benefits of brief community-based lifestyle interventions that promote modest weight loss and increased physical activity are unclear. We assessed the impact of a 13-month lifestyle intervention, the Mobility and Vitality Lifestyle Program (MOVE UP), delivered by community health workers (CHW), on a variety of outcomes, including weight loss, performance-based lower extremity function (Short Physical Performance Battery, SPPB), activity, diet, and health-related quality of life (CDC U48 DP005001). The 32-session behavioral weight management intervention enrolled 303 community-dwelling adults (90.4% of those eligible), who were followed for 12 months (2015-19). Participants completed the program at 26 sites led by 22 CHWs. Participants were age (sd) 67.7 (4.1) and were mostly female (87%). 22.7% were racial minorities. The mean (sd) BMI at baseline was 34.7 (4.7). Median weight loss in the sample was 5% of baseline body weight. SPPB total scores improved by +0.31 units (p < .006), gait speed by +0.04 m/sec (p < .0001), and time to complete chair stands by -0.95 sec (p < .0001). Presenters will assess the effect of MOVE UP on activity, diet, fatigue, and health-related quality of life. A final paper examines implementation of MOVE UP and how site and CHW factors affected outcomes. Findings suggest that promoting healthier eating, weight loss, and physical activity in a community setting is an effective strategy for reducing risk of disability in older adults.

